# *Ecklonia cava* Inhibits Glucose Absorption and Stimulates Insulin Secretion in Streptozotocin-Induced Diabetic Mice

**DOI:** 10.1155/2012/439294

**Published:** 2012-05-08

**Authors:** Hye Kyung Kim

**Affiliations:** Department of Food and Biotechnology, Hanseo University, Seosan 356-706, Republic of Korea

## Abstract

*Aims of study*. Present study investigated the effect of *Ecklonia cava* (EC) on intestinal glucose uptake and insulin secretion. *Materials and methods*. Intestinal Na^+^-dependent glucose uptake (SGU) and Na^+^-dependent glucose transporter 1 (SGLT1) protein expression was determined using brush border membrane vesicles (BBMVs). Glucose-induced insulin secretion was examined in pancreatic *β*-islet cells. The antihyperglycemic effects of EC, SGU, and SGLT1 expression were determined in streptozotocin (STZ)-induced diabetic mice. *Results*. Methanol extract of EC markedly inhibited intestinal SGU of BBMV with the IC_50_ value of 345 *μ*g/mL. SGLT1 protein expression was dose dependently down regulated with EC treatment. Furthermore, insulinotrophic effect of EC extract was observed at high glucose media in isolated pancreatic *β*-islet cells *in vitro*. We next conducted the antihyperglycemic effect of EC in STZ-diabetic mice. EC supplementation markedly suppressed SGU and SGLT1 abundance in BBMV from STZ mice. Furthermore, plasma insulin level was increased by EC treatment in diabetic mice. As a result, EC supplementation improved postprandial glucose regulation, assessed by oral glucose tolerance test, in diabetic mice. *Conclusion*. These results suggest that EC play a role in controlling dietary glucose absorption at the intestine and insulinotrophic action at the pancreas contributing blood glucose homeostasis in diabetic condition.

## 1. Introduction

Diabetes is one of the most prevalent and serious metabolic diseases worldwide. It is characterized by chronic hyperglycemia, impairment of insulin secretion from pancreatic *β*-cells, and insulin resistance in peripheral tissues. One of the therapeutic approaches for decreasing postprandial hyperglycemia is to retard the absorption of glucose by inhibition of carbohydrate-hydrolyzing enzymes such as *α*-amylase and *α*-glycosidase. However, they are not able to prevent glucose absorption when glucose itself has been ingested. Hence, the direct inhibition of intestinal glucose absorption could represent a novel mechanism for an antidiabetic drug.

Intestinal glucose absorption is thought to be regulated by the Na^+^-dependent glucose transporter 1(SGLT1) at the apical membrane of the intestinal epithelia [1]. It has been shown in diabetic animals and humans that the capacity of the small intestine to absorb glucose increases at the brush border membrane vesicles (BBMVs) due to the enhanced activity and abundance of SGLT1 [2, 3].

In order to prevent or delay the progression of diabetes, insulin should be sufficiently secreted to compensate for insulin resistance in peripheral tissues. Thus, one of the mechanisms for the antidiabetic agents requires the characteristics of insulinotrophic action from pancreatic cells [4].

Marine algae have been identified as rich sources of structurally diverse bioactive compounds with great pharmaceutical and biomedical potential. The brown algae, *Ecklonia cava* (EC), are abundant in the south-west coastal region of Japan and Korea. It has been reported that EC extract has numerous biological activities including antioxidative, radical scavenging, immunomodulatory, and antimutagenic activities [5–8]. Recently, Lee et al. demonstrated that polyphenol isolated from EC inhibit *α*-amylase and *α*-glycosidase activities and alleviate postprandial hyperglycemia in streptozotocin- (STZ-) induced diabetic mice [9]. However, the effect of EC on intestinal glucose uptake or insulinotrophic action has not been examined. In this study, we investigated the effects of EC on intestinal Na^+^-dependent glucose uptake, SGLT1 protein expression, and insulin secretion in pancreatic islets. Furthermore, the antihyperglycemic effects of EC in STZ-induced diabetic mice were also evaluated.

## 2. Materials and Methods

### 2.1. Preparation of EC Powder and Extract

 EC was obtained from a local market in Seosan, Republic of Korea. Fresh EC was washed, dried, and ground into powder. The EC powder was used for the *in vivo* experiment, and EC extract was used for the *in vitro* experiment. The dried powder was extracted three times with ten volumes of methanol at room temperature for 24 hour. The combined extracts were centrifuged, filtered, concentrated under vacuum, lyophilized, concentrated to 1 mg/mL with deionized water, and subsequently used for the experiment.

### 2.2. BBMV Isolation

 BBMVs were prepared using a previously described method with some modification [10]. All subsequent isolation steps were performed at 4°C. Male ICR mice (8-week old, Joongang Lab Animal Co., Korea) jejunal mucosal scrapings were suspended in 10 mM HEPES/Tris buffer (pH 7.5), containing 300 mM mannitol and 300 mM MgCl_2_, and homogenized in a glass-Teflon homogenizer (Glass-Col, Terre Haute, IN, USA) for 2 min at 3,000 rpm. The mixture was stirred for 2 min and centrifuged at 15,000 rpm for 15 min at 4°C. The supernatant was centrifuged at 30,000 rpm for 45 min. The resulting pellets were resuspended in 10 mM HEPES/Tris buffer containing 300 mM mannitol (pH 7.5) to a final protein concentration of 10 mg/mL and stored in liquid nitrogen until use. The degree of purity in BBMV was routinely assessed by the enrichment of alkaline phosphatase (ALP) in the finally prepared BBMV compared to the homogenate of intestinal scrapings. ALP activity was determined with ALP assay kit (Yeongdong Pharmaceutical Co., Seoul, Korea). The specific ALP activities of mucosal homogenate and BBMV suspension were 1.21 ± 0.09 and 6.21 ± 0.48 units/mg protein, respectively, exhibiting 5-fold enrichment in final BBMV fraction. The amount of protein in the BBMV was measured by the Bradford method [11].

### 2.3. Na^+^-Dependent Glucose Uptake

 Measurement of Na^+^-dependent glucose uptake by BBMV was determined by incubating 150 *μ*L BBMV suspension with 850 *μ*L uptake buffer containing 100 *μ*M 2-NBDG (Molecular Probes, Grand Island, NY, USA) and EC extract (0.1 mg/mL) at 37°C for 15 min in a shaking water bath. The uptake reaction was stopped by centrifuging for 20 min at 15,000 rpm, and the BBMV pellet was washed with stop buffer. The uptake and stop buffers were 10 mM HEPES/Tris (pH 7.5) containing 150 mM NaCl and 300 *u*M phlorizin (Sigma Co., St. Louis, USA), respectively. Glucose uptake was measured by detecting fluorescence intensity of 2-NBDG with a spectrofluorometer (excitation: 485 nm, emission: 535 nm). The difference between the glucose uptakes in the presence of Na^+^ and phlorizin represents the Na^+^-dependent glucose uptake by SGLT1.

### 2.4. SGLT1 Expression

 Glutaraldehyde was added to Immune well plate and incubated at 37°C for 1 hour. After washing the plate, an antigen of diluted BBMV membrane protein (100 *μ*L/well) and EC were added and incubated at 37°C for 2 hours. After washing the plate, the coated plates were blocked with blocking solution (0.5% casein) at 37°C for 1 hour. Primary antibody incubation was conducted with 100 *μ*L of the SGLTI polyclonal antibody (Chemicon International Inc., Temecula, CA, USA; diluted 1 : 1000 in 0.5% casein) at 37°C for 2 hour. Secondary antibody incubation was conducted with 100 *μ*L of the anti-rabbit IgG conjugated to horseradish peroxidase (diluted 1 : 2000 in 0.5% casein) at 37°C for 1 hour. For substrate incubation, 200 *μ*L of substrate solution (TMB 10 mg/L DMSO, 3% H_2_O_2_, 50 mM sodium acetate buffer, pH 5.1) was added to each well and reacted for 15 min. The enzymatic reaction was stopped by adding 50 *μ*L of 1 M H_2_SO_4_ to each well. The absorbance was determined at 450 nm using automated microplate reader (Model 550, BIO-RAD Laboratories, Philadelphia, PA, USA).

### 2.5. Insulin Secretion

 Pancreatic islet cells were isolated from male mice by collagenase digestion [12]. Twenty islets were preincubated in Krebs-Ringer the bicarbonate (KRB) buffer, pH 7.4, supplemented with serum albumin (3 mg/mL) and glucose (3 mM) for 30 min at 37°C under humidified atmosphere of 5% CO_2_. The islets were treated with EC extract (50 *μ*g/mL) in 3, 8, and 16 mM glucose KRB buffer for 60 min at 37°C. Insulin concentration in each medium was determined using an ELISA procedure (Boehringer Mannheim Diagnostics, Germany).

### 2.6. Animal Study

 Male ICR mice (8-week old) were housed in plastic cages under temperature- (24 ± 2°C) and light- (12-hour light/dark cycle) controlled conditions with constant humidity (55 ± 5%). The study has been carried out along the Korea National Institutes of Health Guidelines on the care and use of laboratory animals and approved by Hanseo University. The mice were randomly divided into four groups (*n* = 10); normal control (NC), normal mice fed EC powder (NE), diabetic control (DC), and diabetic mice fed EC powder (DE). Normal and diabetic control mice were fed AIN-93-based semipurified standard diet, and experimental groups were supplemented with EC powder (3%, w/w). Diabetes was induced by a single intraperitoneal injection of STZ (Sigma, St. Louis, USA; 95 mg/kg in citrate buffer, pH 4.5). NC group received the buffer only. Tail bleeds were performed 24 hour after injection, and animals with blood glucose concentrations above 300 mg/dL were considered to be diabetic and used in this study.

### 2.7. Oral Glucose Tolerance Test and Intestinal BBMV Glucose Uptake

 At the end of 4 weeks of experimental period, mice were fasted overnight and administered with glucose (1.5 g/kg). Blood samples were collected from the tail at various time points (0~90 min) after glucose loading, and blood glucose levels were measured by one-touch basic glucose measurement system (Lifescan Inc., Milpitas, CA, USA). Mice were killed by decapitation immediately after 90-min blood samples were taken. Preparation of intestinal BBMV, Na^+^-dependent glucose uptake, and plasma insulin concentrations were determined as described above.

### 2.8. Statistical Analysis

 Results were presented as mean ± SE, and significant differences (*P* < 0.05) between groups were analyzed by ANOVA followed by Tukey's post hoc test. The 50% inhibitory concentration (IC_50_) value was calculated using Prism program (GraphPad Software Inc., La Jolla, CA, USA).

## 3. Results

### 3.1. Na^+^-Dependent Glucose Uptake and SGLT1 Expression in BBMV

**Figure 1:**
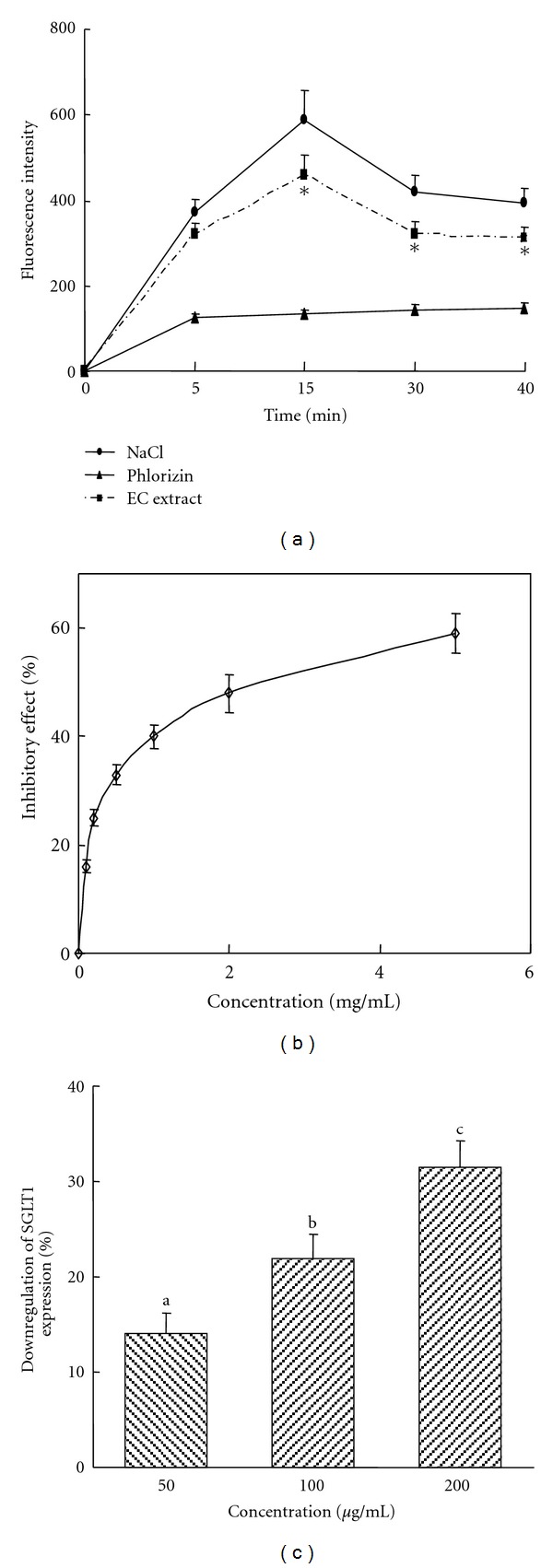
Effects of *Ecklonia cava* (EC) on Na^+^-dependent glucose uptake and SGLT1 expression. (a) Time course effects. *Effect of EC (100 *μ*g/mL) at *P* < 0.05. (b) Concentration-dependent inhibitory effects. Data are expressed as the % of inhibitory activity compared to control group. Glucose uptake was measured with 150 mM NaCl or 300 *μ*M phlorizin to evaluate the sodium dependency of the glucose uptake. (c) Downregulation of SGLT1 expression by EC treatment. SGLT1 abundance was determined by ELISA analysis. ^a–c^Different letters are significantly different at *P* < 0.05. Each value is the mean ± SE of three different experiments.

 The intestinal glucose uptake inhibitory activity of EC extract was determined by *in vitro* model of BBMV using 2-NBDG. Na^+^-dependent 2-NBDG uptake by normal mice intestinal BBMV was time- and concentration-dependent and almost linear up to 15 min and 200 *μ*g/mL (Figures 1(a) and 1(b)). The uptake of 2-NBDG into the intestinal BBMV showed a typical overshoot reaching its peak at 15 min (Figure 1(a)). The overshoot was not observed in the presence of phlorizin, a SGLT1 inhibitor. These results suggest that intestinal glucose uptake mediated by SGLT1 actively occurs after glucose ingestion reaching its peak shortly, and SGLT1 inhibitor abolished the active uptake after glucose ingestion. Furthermore, in the presence of phlorizin, glucose uptake was very low showing about 22% of the total glucose uptake at 15 min indicating that BBMV used SGLT1 as a major mechanism for glucose uptake. EC extract at 100 *μ*g/mL significantly suppressed the Na^+^-dependent glucose uptake into the BBMV in the entire uptake profile (Figure 1(a)). The inhibitory effects were 14, 22, 24, and 22% at 5, 15, 30, and 40 min incubation, respectively. The Na^+^-dependent glucose uptake inhibitory activity of BBMV with various concentrations of EC extract exhibited a dose dependency with IC_50_ at 345 ± 54 *μ*g/mL (Figure 1(b)).

The abundance of SGLT1, measured by ELISA analysis, was downregulated in a dose-dependent manner and showed 31.4% reduction with 200 *μ*g/mL EC treatment (Figure 1(c)).

### 3.2. Glucose-Induced Insulin Secretion in *β*-Islet Cells

**Figure 2:**
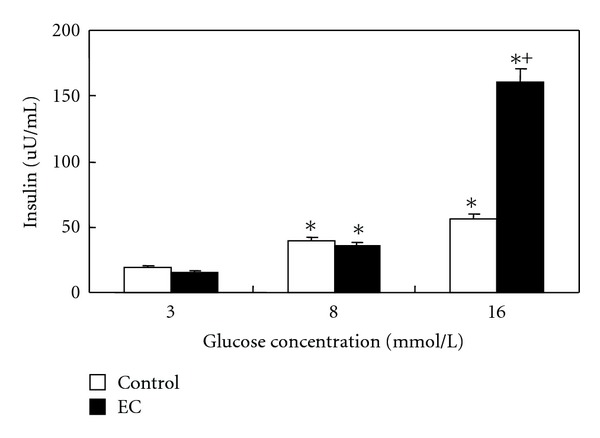
Glucose-stimulated insulin secretion in pancreatic islet cells. The incubations were performed with or without EC extract (50 *μ*g/mL) in 3, 8, or 16 mM glucose media. *Significant effect of media glucose concentration and ^+^significant effect of EC treatment at *P* < 0.05.

Figure 2 shows the stimulatory effect of the EC extract on insulin secretion in the presence of glucose (3~16 mM). Glucose induced a dose-dependent increase of insulin secretion from pancreatic *β*-islet cells in both control and EC-treated group. In the presence of 16 mM glucose, insulin secretion increased by 2.9- and 10.4-fold compared to the 3 mM glucose media in control and EC-treated group, respectively. EC extract fails to stimulate insulin output in 3~8 mM glucose media. However, EC extract significantly boosted insulin secretion in 16 mM glucose media exhibiting 2.8-fold increase as compared with control group.

### 3.3. Intestinal Glucose Uptake in Diabetic Mice

 To validate the *in vitro* results, the antihyperglycemic effects of EC supplementation were investigated in STZ-induced diabetic mice. Induction of diabetes caused significant weight loss resulting in negative body weight gain, whereas EC consumption for 4 weeks ameliorated weight loss (Table 1). The Na^+^-dependent glucose uptake by BBMV prepared from diabetic mice was increased by 39.7% compared with normal mice, and EC supplementation reduced the Na^+^-dependent glucose uptake to near normal value. In normal mice, EC supplementation slightly (14.8%) reduced the Na^+^-dependent glucose uptake without statistical significance. SGLT1 expression in BBMV was 2.6-fold increased in diabetic mice compared to normal mice. Consumption of EC reduced the SGLT1 protein expressions by 17% and 34% in normal and diabetic mice, respectively, confirming the *in vitro* results. The fasting blood glucose concentrations were markedly decreased by EC supplementation in diabetic mice without any significant differences in normal mice. Plasma insulin concentrations were markedly reduced in diabetic mice, and EC supplementation significantly increased the plasma insulin level reaching 75% of the normal insulin level. However, EC supplementation did not affect the plasma insulin concentrations in normal mice.

**Figure 3:**
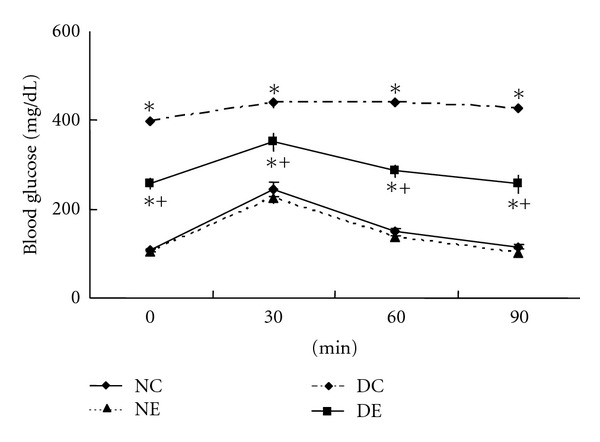
Effect of *Ecklonia cava* (EC) on oral glucose tolerance. Glucose (1.5 g/kg) was administrated at time zero after 12 hour fasting. Normal control mice (NC); normal mice supplemented with EC powder for 4 weeks (NE); STZ-diabetic control mice (DC); diabetic mice supplemented with EC powder (DE). Values are mean ± SE of 10 mice in each group. *Significantly different from normal mice and ^+^significant effect of EC treatment at *P* < 0.05.

The oral glucose tolerance test (OGTT) can be used to evaluate blood glucose homeostasis and also indirectly evaluate glucose absorption. As shown in Figure 3, glucose load in normal mice produced rapid increase in blood glucose levels from 108 ± 4 to 245 ± 15 mg/dL at 30 min and returned to baseline values within 90 min. In contrast, STZ-induced diabetic mice demonstrated basal hyperglycemia (399 ± 14 mg/dL) which remained above 400 mg/dL during all time points determined. The peak increase in serum glucose concentrations in diabetic mice was observed after 60 min of glucose treatment, while that of normal mice observed after 30 min, indicating delayed glucose homeostasis in diabetic mice. Consumption of EC powder slightly reduced fasting blood glucose level in normal mice without any statistical significance. However, EC supplementation in diabetic mice dramatically decreased fasting blood glucose level, and postprandial glucose tolerance showed definite improvement exhibiting similar pattern as normal mice with peak increase at 30 min.

## 4. Discussion

The importance of postprandial glucose control in the development of diabetic complications is widely recognized based on many epidemiological studies. Several inhibitors of *α*-amylase or *β*-glucosidase were proposed to control postprandial hyperglycemia, but the inhibitors of these enzymes are not able to prevent glucose absorption when glucose itself has been ingested. Hence, it might be important to inhibit intestinal glucose absorption as well as glucosidase or amylase activity for the regulation of postprandial blood glucose level.

The capacity of the small intestine to absorb glucose increases in patients with type 2 diabetes and in experimentally induced diabetic animals as a consequence of the enhanced activity and abundance of SGLT1 [2, 3] suggesting SGLT1 as a potential target of drug development for glycemic control in diabetic patients.

In the present study, we conducted an *in vitro* study to examine the effects of EC on intestinal glucose uptake using BBMV. The results revealed that EC reduced SGLT1 activity, assessed by Na^+^-dependent glucose uptake and SGLT1 protein expression in BBMV. Furthermore, the insulinotrophic effect of EC extract was observed at high glucose (16 mM) environment. Compared to the results at 3 mM media glucose, insulin secretion at 16 mM glucose with EC extract increased by 10.4-fold. This is consistent with the observation that glucose acts synergistically with antidiabetic plant to promote insulin secretion [13]. The elevated insulin secretion with EC treatment at high glucose environment agrees with *in vivo* results in Table 1, thus strengthening the evidence that the EC acts as a stimulator of insulin secretion. Diabetic mice (blood glucose > 20 mM) markedly increased plasma insulin concentration by 192% while Na^+^-dependent glucose uptake was reduced by 22% in response to EC supplementation. These results suggest that the relative contribution of increased islet cell insulin secretion is more potent than reduced intestinal Na^+^-dependent glucose uptake for the regulation of blood glucose level in EC-supplemented diabetic mice. Therefore, it is significant that the EC extract could potentiate insulin secretion at higher glucose concentration, which could be useful in situations where chronic hyperglycemia decreases the sensitivity of *β*-islet cells to glucose-induced insulin secretion [14].

We subsequently examined the *in vivo* effect of EC on glucose uptake by BBMV prepared from STZ-diabetic mice fed EC diet. Recent studies have shown that modifications of systemic glycemia in OGTT reflect the activity of the intestinal glucose transporter SGLT1 [15]. STZ-induced diabetic mice exhibited severe hyperglycemia with increased Na^+^-dependent glucose uptake activity and SGLT1 expression in intestinal BBMV compared with normal mice. EC consumption reduced intestinal Na^+^-dependent glucose uptake and SGLT1 expression in diabetic mice resulting in improvement of OGTT and blood glucose level. The blood glucose and insulin levels in EC supplemented mice were not completely normalized whereas the intestinal Na^+^-dependent glucose uptake was normalized, again suggesting the importance of pancreatic insulin secretion for the regulation of blood glucose homeostasis. The enhancement of intestinal glucose uptake in diabetic mice might be attributed to several factors, such as an increase in mucosal mass, an increase in the turnover of the transporter, and/or an increase in the number of SGLT1. It has been reported that glucose transporter levels including SGLT1 are elevated in diabetic animals and humans [2, 3].

Administration of EC suppressed the body weight loss in diabetic mice suggesting that EC treatment may beneficially affect the metabolic state in diabetes. Indeed, it has been reported that administration of phlorizin or T-1095, Na^+^-dependent glucose transporter inhibitor, leads to a body weight gain in STZ diabetic rats [16, 17].

Seaweeds contain appreciable amounts of polyphenols, and Ahn et al. [18] reported that the content of polyphenols in EC is about 18.3%. It has been suggested that polyphenols modulate hyperglycemia through various mechanisms. First, glucose absorption from intestine was reduced by inhibiting *α*-glucosidase and/or SGLT1 activity. Catechin [19], flavonoid [20], and quercetin [21] have been reported to inhibit *α*-glucosidase activity via steric hindrance. The influence of polyphenols on glucose transporters has been studied *in vitro* by using intestinal BBMV or everted sacs and Caco-2 cells. The Na^+^-dependent SGLT1-mediated glucose transport was inhibited by tea catechins [22, 23], tannic acids [24], and quercetin monoglucosides [25]. Kobayashi et al. [22] and Shimizu et al. [23] reported that green tea polyphenols, especially epicatechin gallate (ECg), inhibit SGLT1 in a competitive manner interacting as antagonist-like molecules, although ECg itself was not transported via the glucose transporters. Oliveira et al. reported that polyphenol-rich Yerba maté decreased intestinal SGLT1 expression in alloxan-induced diabetic rats [26]. Second, polyphenols increase pancreatic insulin secretion. Soy isoflavonoids (genistein and daidzein) preserved insulin production by the *β*-cells in STZ-induced diabetic mice [27]. EGCG activates IRS2 and AMPK signalling in rat pancreatic *β*-cells [28]. Genistein augments cAMP accumulation and insulin release in MIN6 cells [29]. Therefore, it is obvious that the pancreas is one of the targets of dietary polyphenol bioactivity although no single mechanism has been identified to be responsible for the response. Although the identity of chemical component responsible for the antidiabetic action of EC in present study is unknown, it would be of considerable interest to further elucidate the mechanism and component(s) underlying the action of EC.

In conclusion, this study demonstrates that EC manifests two important antidiabetic properties: the inhibition of intestinal Na^+^-dependent glucose absorption mediated by reduced expression of SGLT1 and the stimulation of insulin secretion in hyperglycemic environment, resulting in improvement of the glucose regulation in diabetic condition. The present findings identify, for the first time to our knowledge, the inhibitory effect on intestinal glucose uptake and insulinotrophic effect of EC.

## Figures and Tables

**Table 1:** Effect of *Ecklonia cava* (EC) on blood glucose regulation.

	NC	NE	DC	DE
Body weight gain (g)	7.8 ± 0.9^a^	8.4 ± 0.6^a^	−5.9 ± 0.7^b^	−2.1 ± 0.4^c^
Glucose uptake^1^	357 ± 28^a^	304 ± 17^a^	499 ± 33^b^	392 ± 22^a,c^
SGLT1 expression^2^	1.00 ± 0.21^a^	0.83 ± 0.22^a^	2.61 ± 0.34^b^	1.72 ± 0.20^c^
Blood glucose (mg/dL)	112 ± 10^a^	109 ± 1^a^	395 ± 13^b^	240 ± 19^c^
Plasma insulin (ng/mL)	1.28± 0.03^a^	1.25± 0.03^a^	0.50 ± 0.01^b^	0.96 ± 0.03^c^

Values are means ± SE of 10 mice in each group. NC: normal control mice; NE: normal mice supplied with EC powder (3%). DC: STZ-mice; DE: STZ-mice supplied with EC. ^1^Na^+^-dependent glucose uptake by BBMV was expressed as fluorescence intensity. ^2^Relative SGLT1 protein expression.

^a–c^Different superscript letter means significantly different at *P* < 0.05.

## Abbreviations

EC: *Ecklonia cava*
SGU: Na^+^-dependent glucose uptake
SGLT1: Na^+^-dependent glucose transporter 1
BBMV: Brush border membrane vesicles
STZ: Streptozotocin
ALP: Alkaline phosphatase.
